# Managing treatment-resistant schizophrenia following clozapine cessation: a case report of xanomeline–trospium and olanzapine combination therapy

**DOI:** 10.3389/fpsyt.2025.1679678

**Published:** 2025-10-21

**Authors:** Nils Sumegi Went, Juan D. Alonso, Rosemarie R. Rigunay, Jason Hershberger, Xiao Xiong You

**Affiliations:** Department of Psychiatry, Brookdale University Hospital and Medical Center, Brooklyn, NY, United States

**Keywords:** treatment-resistant schizophrenia (RTS), clozapine, venous thromboembolism (VTE), pulmonary embolism (PE), xanomeline—trospium, olanzapine

## Abstract

**Background:**

Clozapine is the gold standard for treatment-resistant schizophrenia (TRS) but is limited by rare, serious adverse events. Venous thromboembolism (VTE) and pulmonary embolism (PE) represent particularly challenging complications given their multifactorial pathophysiology, unpredictable recurrence risk, and lack of clear guidance on rechallenge.

**Case presentation:**

A 44-year-old man with TRS and comorbid seizure disorder achieved over 25 years of remission on clozapine (300 mg/day). In November 2023, he developed acute deep vein thrombosis and PE, leading to permanent discontinuation despite long-term tolerability and the absence of conventional risk factors. Abrupt cessation was followed by rapid relapse. Aripiprazole, titrated to 15 mg/day, and subsequent augmentation with olanzapine failed to restore stability. A muscarinic–dopaminergic approach using xanomeline–trospium (Cobenfy™) with olanzapine was initiated, producing marked improvements in positive symptoms, affective expressivity, social engagement, and daily functioning without significant adverse effects.

**Discussion:**

Most clozapine-related VTE/PE cases necessitate discontinuation, though rare continuations under anticoagulation and multidisciplinary oversight have been described. Reviews emphasize inconclusive evidence and the need for individualized risk–benefit assessment. In this case, discontinuation was chosen collaboratively with the family, prompting exploration of alternative mechanisms.

**Conclusion:**

Clozapine-related VTE/PE represents a serious clinical dilemma. This case illustrates the potential of muscarinic–dopaminergic strategies to restore stability in TRS when clozapine is no longer an option.

## Introduction

Treatment-resistant schizophrenia (TRS) affects approximately 20–30% of individuals diagnosed with schizophrenia (1). TRS is typically defined by the persistence of positive symptoms, such as hallucinations and delusions despite adequate trials of at least two antipsychotic medications administered at therapeutic doses. In addition to refractory psychosis, patients frequently exhibit prominent negative symptoms and cognitive impairments, including deficits in attention, working memory, and executive function. These domains represent core features of schizophrenia and are strongly associated with long-term disability, even when positive symptoms are partially attenuated (2, 3). As a result, individuals with TRS often experience increased hospitalization rates, impaired social and occupational functioning, and heightened caregiver burden (1, 4).

Clozapine remains the most effective and evidence-based treatment for TRS, with approximately 30–60% of patients achieving meaningful symptom reduction (4). Its superior efficacy is thought to arise from a unique receptor-binding profile, characterized by weak D_2_ antagonism, higher affinity for D^4^ receptors, and potent antagonism of serotonergic (5-HT_2_A), adrenergic, histaminergic, and muscarinic receptors (5). However, clozapine remains significantly underutilized, with fewer than one-third of eligible TRS patients receiving it. This is largely due to its complex adverse effect profile, which includes agranulocytosis, myocarditis, seizures, and severe metabolic disturbances. Critically, clozapine has also been associated with rare but potentially fatal complications such as venous thromboembolism (VTE) and pulmonary embolism (PE) (2). Overall, up to 76% of patients experience side effects, and approximately 17% discontinue treatment due to adverse events (6).

Although rare, clozapine-associated VTE and PE are serious complications that may occur early in the treatment course or after long-term maintenance. These events can arise independently of dose or established VTE risk factors (7, 8). While most cases are reported within the first 12 weeks of initiation, delayed-onset thromboembolic events underscore the importance of ongoing clinical vigilance (9, 10).

The pathophysiology of clozapine-induced VTE appears multifactorial. Contributing mechanisms may include sedation, weight gain, and metabolic abnormalities that reduce physical activity and promote venous stasis (11, 12). Laboratory studies suggest that clozapine enhances platelet adhesion and aggregation, alters fibrinogen structure, and accelerates thrombus formation, factors that increase thrombogenic potential (13, 14). Additionally, long-term exposure has been associated with elevated antiphospholipid antibodies, which may further predispose to thrombotic events (15).

Collectively, these findings highlight the convergence of clinical, metabolic, and hematologic factors in clozapine-related thrombosis. Recognition of these risks is essential, as the development of VTE or PE frequently necessitates permanent discontinuation of clozapine in patients who have otherwise responded to treatment, leaving clinicians with few effective alternatives.

## Case presentation

A 44-year-old African American man with a long-standing diagnosis of treatment-resistant schizophrenia (TRS) and a comorbid seizure disorder presented on November 25, 2023, with acute swelling and pain in his right leg. His TRS had remained in sustained remission for 26 years while being treated with clozapine (300 mg per day; 100 mg three times a day), which was started in 1998 after multiple failed trials with other antipsychotic medications. He had been functionally stable, living with his mother, who supervised his medication adherence and attended a structured psychiatric day program. Additionally, valproic acid (250 mg twice a day) was continued for seizure prophylaxis.

At presentation to the emergency department, the patient was afebrile with oxygen saturation of 99% on room air. Physical examination revealed swelling, erythema, warmth, tenderness, and firm induration of the right thigh and calf, with visible superficial venous engorgement. Laboratory testing demonstrated leukocytosis (WBC 12.6 × 10³/µL; 61.8% neutrophils) and a markedly elevated D-dimer (>7,650 ng/mL). Blood cultures were obtained, and empiric intravenous cefepime and vancomycin were initiated. CT pulmonary angiography confirmed a right upper-lobe pulmonary embolism (PE) and extensive deep vein thrombosis (DVT), which was corroborated by venous Doppler ultrasound. Clozapine was permanently discontinued due to its known association with VTE. Therapeutic anticoagulation was initiated with enoxaparin (1 mg/kg BID) and later transitioned to apixaban (5 mg BID). The patient was discharged on December 12, 2023, and maintained on aripiprazole 5 mg daily.

Discontinuing clozapine posed a significant therapeutic challenge due to the patient’s long-standing reliance on it for managing symptoms. Aripiprazole was initiated and gradually increased to 15 mg daily by February 2024, following reports from his mother about worsening symptoms. These included thought blocking, persistent suspiciousness, and auditory hallucinations characterized by voices (both male and female), along with safety-seeking behaviors, such as repeatedly closing curtains prompted by persecutory delusions. Due to the ongoing symptoms, the dose of aripiprazole was raised to 20 mg in April 2024.

By October 2024, it was further escalated to 30 mg after staff members at his day program observed pacing, verbal responses to internal stimuli, and persistent hallucinations. Despite full adherence to the medication regimen, the patient continued to experience persecutory delusions, command hallucinations, visual misperceptions, and significant negative symptoms (such as affective flattening, avolition, and social withdrawal) as well as cognitive symptoms (including poor attention and executive dysfunction). These issues led to a marked decline in his daily functioning.

In March 2025, olanzapine 5 mg daily was added to address worsening auditory hallucinations, paranoid ideation, and recurring safety-seeking behaviors. The dose was titrated to 10 mg by April 2025; however, concerns expressed by his family limited further dose escalation. Despite dual therapy with aripiprazole and olanzapine, the patient’s response remained suboptimal.

On April 15, 2025, he was transported via emergency medical services to a Comprehensive Psychiatric Emergency Program (CPEP) for acute psychotic exacerbation and behavioral disorganization, characterized by reactive gesturing, striking doors in response to perceived threats, and verbalizations to internal stimuli.

During inpatient admission (April 15–May 9, 2025), aripiprazole was tapered and discontinued. Xanomeline–trospium a muscarinic M_1_/M^4^ receptor agonist with a non-dopaminergic mechanism was initiated at 50/20 mg BID and titrated to 100/20 mg BID. Olanzapine was maintained at 5 mg in the morning and 10 mg at bedtime. Valproic acid (250 mg BID) and apixaban (5 mg BID) were continued. Supportive bowel regimens (docusate sodium and polyethylene glycol) were used to mitigate anticholinergic side effects. At discharge, the patient denied active hallucinations but demonstrated occasional self-directed speech and reduced verbal spontaneity.

At follow-up on May 20, 2025, the patient’s mother reported moderate psychosocial improvement. He had resumed community outings, participated in virtual religious services, and engaged in leisure activities. His affect was stable, with no reported aggression or emotional dysregulation, although mild paranoid ideation persisted.

By June 3, he acknowledged mild situational depressive symptoms but retained insight. His mother noted renewed suspiciousness and expressed concerns about surveillance-related delusions. Clinical observation confirmed gesturing and verbal responses to perceived threats. Consequently, xanomeline–trospium was increased to 125/30 mg BID.

On June 17, modest improvements were observed in self-directed speech and social reciprocity at a religious event. Although attenuated paranoid ideation remained, there was no recurrence of VTE.

By July 1, the patient resumed regular attendance at his day program, albeit with mild internal preoccupation. Psychoeducation sessions were conducted with caregivers to support a low-expressed-emotion home environment.

By July 14, significant clinical stabilization was evident. The patient was appropriately groomed, with mild psychomotor slowing, coherent speech, only infrequent thought blocking, and notably reduced paranoid ideation. Safety-seeking behaviors were absent, anxiety had decreased, and his affect was more reactive. He demonstrated improved insight, judgment, and social engagement, as well as greater independence in activities of daily living, including self-transportation to church. His mother reported a marked improvement in emotional reciprocity and consistent participation in programming.

On July 29, the patient remained calm, cooperative, and free of hallucinations or paranoid ideation. Both he and his mother denied recent aggression or self-injury. Functional recovery continued with improved interpersonal engagement.

During an August 26 telehealth session, while his mother was hospitalized for a nephrectomy, her niece reported no bizarre behaviors. The patient appeared calm and cooperative, with no safety-seeking behaviors or psychotic symptoms. His mother later noted occasional self-talk, though markedly less frequent and intense than prior to initiating Xanomeline–trospium.

In comparison to the significant symptom relapse observed after discontinuing clozapine and the inadequate response to both aripiprazole monotherapy and the combination of aripiprazole and olanzapine, the introduction of xanomeline–trospium with olanzapine resulted in considerable improvements. These improvements were seen in positive symptoms, emotional expression, social functioning, and caregiver burden, indicating a meaningful clinical and functional recovery in a previously treatment-resistant case of schizophrenia.

## Discussion

This case highlights the clinical complexity of managing TRS following clozapine discontinuation due to a life-threatening adverse event. The patient developed VTE and PE after more than 25 years of remission on clozapine (300 mg/day), despite stable dosing, long-term tolerability, and absence of conventional risk factors. His clinical trajectory illustrates clozapine’s therapeutic paradox. While clozapine remains the most effective antipsychotic for TRS, with unparalleled efficacy against hallucinations, delusions, suicidality, and functional decline, its use is constrained by adverse events comprising agranulocytosis, myocarditis, seizures, and rare but serious thromboembolic complications (16, 17). Although clozapine-associated VTE most often occurs within the first 12 weeks of treatment, delayed presentations have been documented and may occur after years of maintenance therapy (7, 8). Abrupt discontinuation, as in this case, frequently precipitates rapid relapse, consistent with reports that nearly half of patients experience exacerbation within six months of stopping clozapine (18, 19).

Some serious complications, such as agranulocytosis, myocarditis, or seizures, may not represent absolute contraindications to rechallenge. Advances in hematologic monitoring and the use of hematopoietic growth factors have enabled selected patients to safely resume clozapine after agranulocytosis under strict surveillance (20–22). Similarly, rechallenge has been attempted after myocarditis, once cardiac function stabilizes, with gradual titration and close monitoring (23, 24). Seizures, often dose-dependent, can be managed with anticonvulsant co-therapy or dose adjustment. Current guidance emphasizes that clozapine’s unmatched ability to reduce suicidality, hospitalization, and mortality can justify cautious rechallenge when precipitating factors are reversible or manageable (25).

By contrast, the occurrence of VTE or PE presents a more formidable dilemma. Multiple reports confirm an association between clozapine and thromboembolism, implicating metabolic effects, immobility, platelet activation, fibrinogen abnormalities, and proinflammatory mechanisms (8–15, 30). However, guidance on rechallenge remains limited. Systematic reviews emphasize inconclusive evidence, recommending individualized risk–benefit assessment rather than automatic discontinuation (7, 26, 27, 31). Rare instances of safe continuation have been reported under anticoagulation and multidisciplinary oversight (28, 29, 31); however, most published cases describe permanent cessation due to the risk of recurrence (7, 26, 27). The heterogeneity of outcomes underscores the absence of a uniform strategy, highlighting the importance of case-by-case decision-making (32). In our case, permanent discontinuation was chosen collaboratively with the patient’s family, reflecting the unpredictability of recurrence and lack of viable preventive measures.

Following clozapine cessation, aripiprazole was selected due to its favorable safety profile, including a lower thromboembolic risk compared to clozapine and olanzapine (32, 33). Despite titration to 30 mg/day, the patient remained psychotic with worsening negative and cognitive symptoms, consistent with reports of limited efficacy in entrenched illness and dopamine super sensitivity. Augmentation with olanzapine was attempted to broaden receptor coverage and approximate the multimodal activity of clozapine. However, while some evidence supports this combination, clinical benefits are typically modest and inconsistent (34–36). In this case, persistent hallucinations and delusions confirmed the limitations of dopaminergic augmentation in true TRS.

Given these limitations, attention turned to non-dopaminergic strategies. Xanomeline–trospium, a muscarinic M_1_/M^4^ receptor agonist–antagonist combination approved in 2024, targets both glutamatergic and dopaminergic circuits implicated in the pathophysiology of schizophrenia (37). Xanomeline acts centrally as a muscarinic receptor agonist, whereas trospium, a peripherally restricted anticholinergic, reduces unwanted cholinergic effects such as gastrointestinal distress and hypersalivation (38). In the EMERGENT trials, xanomeline–trospium produced significant reductions in both positive and negative symptoms (Cohen’s d ≈ 0.65), with a more favorable safety profile than conventional D_2_ receptor antagonists, particularly with respect to metabolic, hematologic, and extrapyramidal side effects (39). Although patients with treatment-resistant schizophrenia were excluded from these studies, emerging case series report symptomatic and cognitive improvements in clozapine-intolerant populations, further supporting the therapeutic potential of xanomeline–trospium in difficult-to-treat cases (40).

In our patient, the introduction of xanomeline–trospium combined with olanzapine yielded substantial gains in positive symptoms, affective expressivity, community engagement, and functional autonomy. Importantly, the combination did not produce additive cognitive, autonomic, or gastrointestinal side effects, possibly reflecting pharmacodynamic offset between xanomeline’s central muscarinic agonism and olanzapine’s antagonism, with trospium further limiting peripheral burden. Compared to the severe deterioration following clozapine withdrawal and the inadequate response to dopaminergic therapy, this regimen resulted in clinically meaningful recovery, reduced caregiver strain, and improved quality of life.

While this outcome is encouraging, it represents a single case and should be interpreted cautiously. A systematic evaluation is needed to establish the safety, tolerability, dosing strategies, and long-term outcomes of muscarinic–dopaminergic therapy in TRS. Given the risk of cumulative anticholinergic burden, particularly when combined with olanzapine, these regimens should be employed judiciously with individualized risk–benefit assessment, multidisciplinary collaboration, and close monitoring.

## Conclusion

This case illustrates the clinical challenges of treating TRS when clozapine is discontinued due to severe thromboembolic complications. Clozapine withdrawal creates a significant therapeutic gap. In this patient, aripiprazole monotherapy and aripiprazole-olanzapine combination therapy were ineffective, despite dose optimization and psychosocial support. Transitioning to xanomeline–trospium in combination with olanzapine resulted in substantial clinical and functional improvements, the resolution of positive symptoms, an increased affective range, better social participation, and restored autonomy, with no additive adverse effects. These findings suggest that muscarinic-dopaminergic pharmacologic strategies may be useful for carefully selected patients with TRS, who cannot tolerate or continue clozapine. Prospective studies are needed to evaluate their role as second-line therapies, optimize treatment protocols, and assess long-term safety and functional outcomes.

## Data Availability

The original contributions presented in the study are included in the article/supplementary material. Further inquiries can be directed to the corresponding author.
